# Evolution of specialized toxin arsenals in a bacterial symbiont of arthropods

**DOI:** 10.1093/ismejo/wraf174

**Published:** 2025-08-11

**Authors:** Logan D Moore, Matthew J Ballinger

**Affiliations:** Department of Biological Sciences, Mississippi State University, Mississippi State, MS 39762, United States; Department of Biological Sciences, Mississippi State University, Mississippi State, MS 39762, United States

**Keywords:** ribosome-inactivating proteins, toxins, symbiosis, defense, parasitism, Spiroplasma

## Abstract

Bacteria commonly deploy toxic proteins that act with specificity on target molecules to support invasion and improve survival in competitive environments. Many toxin-encoding bacteria have evolved into host-associated defensive partnerships, in which they use toxins to improve host survival during infection. The stability of these relationships requires that symbiont toxins target diverse parasites while minimizing damage to the host. We investigate the specificity of a group of ribosome-targeting toxins (ribosome-inactivating proteins) encoded by heritable *Spiroplasma* symbionts that contribute to defense against parasite infection in fruit fly hosts. Using *Escherichia coli* to express five divergent copies of this toxin, we show that distantly related members of the family all retain the ability to inactivate ribosomes by adenine cleavage at the α-sarcin/ricin loop, the enzymatic hallmark of RIPs. However, when exposed to live insect and fungal cells, ribosome inactivation varies across the five toxins, suggesting cellular recognition or localization play a role in target specificity. To identify toxin domains required for specificity, we removed rapidly evolving “accessory” domains from two toxins. Both truncated toxins exhibit significantly increased activity on purified ribosomes *in vitro*, suggesting one role of accessory domains is to reduce toxicity, which may help protect hosts from collateral damage. One of the truncated toxins also showed significantly reduced inactivation of cellular ribosomes *in vivo*, indicating a role for accessory domains in cell specificity. Together, these data reveal a mechanism for symbiont discrimination between hosts and parasites and highlight how dynamic toxin evolution can contribute to stability and novelty in defensive symbiosis.

## Introduction

Protein toxin genes are often under intense selection pressure due to their key roles in infection and immunity, and their co-evolution with target molecules [1–3]. As a result, protein toxin genes exhibit patterns of rapid evolution across biological kingdoms [4–9]. One frequently observed molecular evolutionary process is expansion of toxin-encoding genes into multicopy gene families, perhaps for the initial benefit of increased toxin production [10]. Mutations in expanded toxin gene families may result in the inactivation of some gene duplicates (pseudogenization) whereas in others they may support split functions (subfunctionalization) or new functions (neofunctionalization). Expanded and diversified protein toxin families can also be further shaped by recombination and lateral transfer, often resulting in functional diversity with adaptive benefits [4, 11–14]. This functional diversity is exemplified by bacterial protein toxins and is consequential for human interests. For instance, the bacterium *Bacillus thuringiensis* is a crucial line of defense against agricultural pests due to the nearly 700 insecticidal Cry toxin variants that they can produce [9, 15]. Insect susceptibility to a given *B. thuringiensis* strain is strongly influenced by the Cry toxin variants it encodes. Given the role of bacterial toxin functional diversity in human health, agriculture, and ecology [8, 9, 16–18], understanding how this diversity arises, evolves, and affects infection phenotypes is key to predicting their future implications and downstream applications.

*Spiroplasma* is a genus of small, helical, and cell wall-less bacteria infecting upwards of 7% of terrestrial arthropods [19]. Members of the genus are classified into three clades; the Citri-Chrysopicola-Mirum (hereafter referred to as Citri), Ixodetis, and Apis clades [20]. Maternally transmitted *Spiroplasma* are currently known in the Citri clade and the Ixodetis clade, and these species often encode toxins and toxin-related genes from multiple protein families [18, 21, 22]. Type 1 ribosome-inactivating protein (RIP) toxins are among the most expansive and diverse toxin families within *Spiroplasma*. RIP toxins are a class of plant-originating toxins including ricin from the castor bean plant and Shiga-like toxin from pathogenic Shiga toxin-producing *E. coli* [23]. RIPs can be potent toxins due to the irreversible damage (i.e. depurination) they inflict on ribosomes and the catalytic rate at which they inflict it; though this depends largely on their ability to surpass cellular barriers to access those targets [24, 25]. *Spiroplasma* RIP toxins are believed to contribute to defensive phenotypes whereby *Spiroplasma* protects its insect host against multicellular parasites [26, 27]. Defense is best studied in members of the Citri clade that infect *Drosophila* hosts, including the strains MSRO (melanogaster sex ratio organism), *sHyd*, and *sNeo* which infect *Drosophila melanogaster, D. hydei,* and *D. neotestacea*, respectively. All three strains defend their hosts against parasitoid wasps including members of the genera *Asobara*, *Leptopilina*, and *Ganaspis* [28–30]. In addition, *Spiroplasma* strain *sNeo* protects its host against sterilization by the parasitic nematode *Howardula aoronymphium* [31].

**Figure 1:**
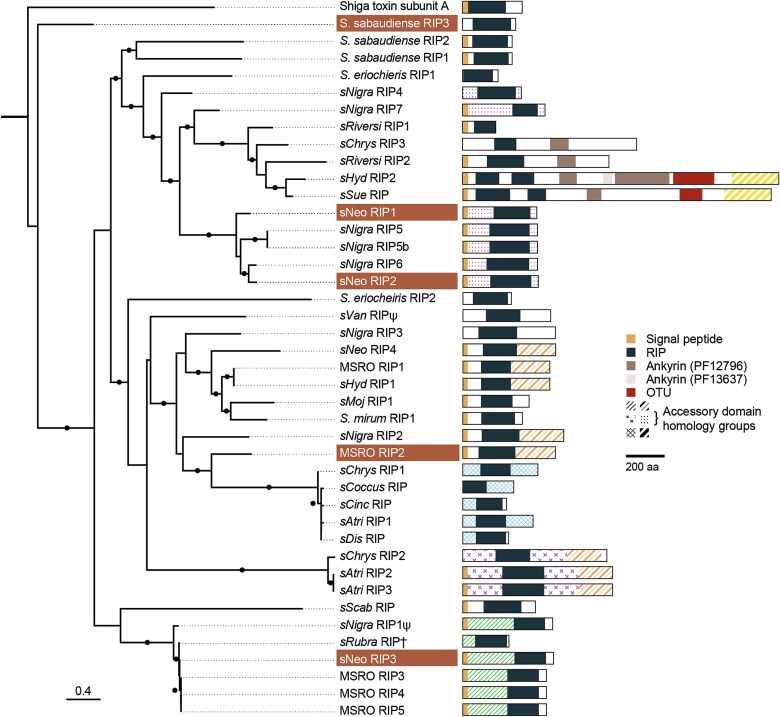
RIP diversity and architecture. A maximum likelihood phylogeny of RIPs constructed from an amino acid alignment of the RIP domain. Beside each branch label is a scaled representation of the domain architecture of the RIP (signal peptide domains are not to scale). BLAST-inferred homology between accessory domains of different RIPs is shown as tiled patterning. Regions in which the presence of a homologous accessory domain is not strongly supported are shown in white. The Greek letter psi (Ψ) indicates a pseudogene and † indicates an incomplete sequence due to contig length. Black points indicate branch support values >0.60. RIP toxins purified and used in this study are highlighted. Phylogenetic scale represents substitutions per site.

*Spiroplasma* strains can encode multiple RIP toxins. Some maintain as many as eight divergent copies [32] whose phylogenetic relationships indicate a history of duplication, horizontal gene transfer, and recombination events [18, 32]. *Spiroplasma* RIPs vary greatly in size, sequence similarity, and structure. For instance, RIP domain-possessing proteins range in length from <300 amino acids (aa) to >1500 aa. RIP domains can share <30% sequence similarity and are often flanked by highly divergent or nonhomologous accessory domains of varied or unknown functions (Fig. 1). This striking toxin diversity is hypothesized to contribute to defensive outcomes supported by individual *Spiroplasma* strains. For example, *H. aoronymphium* sterilizes several species of *Drosophila* [33], but fecundity is restored by co-infection with *Spiroplasma* strain *sNeo* [31] but not MSRO or *sHyd*. Additionally, nematode motherworms exhibit high levels of ribosome depurination when exposed to *sNeo* but not MSRO *in vivo* [32]. These observations are complemented by the presence of two RIP toxins encoded by *sNeo* (sNeo RIP1 and sNeo RIP2) that are distantly related to RIP toxins encoded by the strains lacking nematode defense, MSRO and *sHyd* (Fig. 1), suggesting specificity may be determined in part by interactions between individual RIP toxins and their target organisms. Additional evidence of RIP specificity is demonstrated by host–RIP interactions. Toxins of defensive bacteria must balance potency toward enemies against harm to the host. Indeed, some *Drosophila* hosts of *Spiroplasma* symbionts show signs of RIP-mediated health impacts, e.g. reduction in host hemocyte count and host lifespan [34]. Even still, RIP activity and fitness costs are significantly lower in host flies compared to parasites [27]. RIP toxins are secreted freely into the host hemolymph, suggesting biological barriers (e.g. cellular membranes, intracellular trafficking, degradation pathways) differentially prevent specific RIPs from accessing nontarget cells. Other defensive symbionts may balance a similar tightrope of potency and specificity. For instance, *Hamiltonella defensa* uses diverse phage-encoded protein toxins to defend its aphid hosts against parasitoid wasps [35] and a strain of *Pseudomonas* produces a translation-inhibiting amide, pederin, to defend its beetle host from predation [36]. Thus, in addition to supporting critical processes in antagonistic microbe-host interactions, toxins confer phenotypes that help to stabilize beneficial associations across ecological and evolutionary timescales.

In this study, we investigate the functional diversity of *Spiroplasma* RIP toxins by purifying a panel of RIPs representative of toxin diversity across the genus. We demonstrate conservation of N-glycosidase activity across the toxin family and show that individual strains maintain multiple active RIP toxins. We also demonstrate that the activity of individual RIP copies is differentially influenced by target identity (i.e. ribosome or cell type), suggesting that the emergence and persistence of genetic variation in this gene family has clear functional implications. Finally, we explore the role of hyperdiverse accessory domains flanking the RIP toxin domain using truncation experiments and find evidence these domains can inhibit RIP activity against free ribosomes but also play crucial roles for RIP activity in live cells, highlighting a potential evolutionary tradeoff between toxin activity and target specificity in the *Drosophila*–*Spiroplasma* mutualism.

## Materials and methods

### Cell stocks

*Aedes albopictus* (C7–10) cells were maintained in minimum essential media (MEM) (Gibco) supplemented with 10% fetal bovine serum (FBS) (Corning) at 28°C and 5% CO_2_. *Drosophila melanogaster* (S2) cells were maintained in Schneider’s media (Gibco) with 10% fetal bovine serum (FBS) at room temperature. *Saccharomyces cerevisiae* (HA0) cells were grown in YPD liquid media at 30°C and stored with 25% glycerol (Invitrogen) at −80°C. *E. coli* (New England Biolabs SHuffle T7) cells were grown in LB liquid media at 30°C and stored with 25% glycerol at −80°C.

### Purification and truncation of *Spiroplasma* RIP toxins

**Figure 2:**
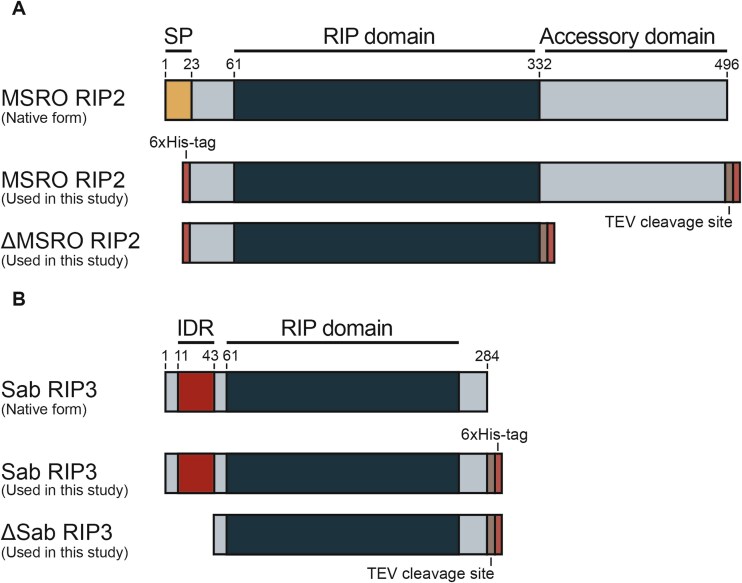
Protein variants of MSRO RIP2 and Sab RIP3 toxins. The C-terminal accessory domain of MSRO RIP2 and the N-terminal accessory domain of Sab RIP3 were removed to characterize their function in toxin specificity. (A) A scaled protein representation of the multiple variants of MSRO RIP2 toxin. The top representation shows MSRO RIP2 as it would be produced by *Spiroplasma* prior to secretion*.* The middle representation shows the MSRO RIP2 used in this study which lacks a signal peptide and has been adapted with 6xHis-tags on both the N-terminal and C-terminal ends. The bottom representation shows the truncated version of MSRO RIP2 (ΔMSRO RIP2) which lacks an accessory domain. HMMER software was used for domain annotation. (B) A scaled protein representation of the multiple variants of Sab RIP3 toxin. The top representation shows Sab RIP3 as it would be produced by *Spiroplasma.* The middle representation shows the Sab RIP3 used in this study which has been adapted with a 6xHis-tag on the C-terminal end. The bottom representation shows the truncated version of Sab RIP3 (ΔSab RIP3) which lacks an accessory domain. HMMER software was used to determine domain annotations.

Five *Spiroplasma* RIP toxins were selected for recombinant expression and purification including sNeo RIP1–3, Sab RIP3, and MSRO RIP2 (Fig. 1). Signal peptides were detected using SignalP 6.0 and removed at the predicted cleavage site. The RIP sequences underwent codon optimization for expression in *E. coli*. A TEV cleavage site and a 6-His tag were added to the C-terminal ends of RIP toxins. These gene constructs were then inserted into either a pBR322 plasmid or a pET15-b plasmid. SHuffle T7 Express Competent *E. coli* were transformed with these RIP-bearing plasmids and toxin expression was induced with IPTG. Expressed *Spiroplasma* RIP toxins were purified on a HisPur Ni-NTA Resin column and stored at −80°C. Sab RIP3 and MSRO RIP2 accessory domains were truncated using a Q5 Site-directed mutagenesis kit to produce ΔSab RIP3 and ΔMSRO RIP2 (Fig. 2). See supplementary methods for more specific information regarding *Spiroplasma* RIP toxin purification and truncation.

### Ribosome isolation

Ribosomes were isolated from three eukaryotic cell lines including *A. albopictus* (C7-10), *D. melanogaster* (S2), and *S. cerevisiae* (HA0). All cell types were lysed, and the lysate was overlayed on top of a sucrose cushion. Ribosomes were isolated via passage through the sucrose cushion with ultracentrifugation. Ribosome pellets were resuspended in a ribosome storage buffer and stored at −80°C. See supplementary methods for more specific information regarding ribosome isolation.

### Ribosome-inactivating protein assays on rabbit reticulocyte and isolated ribosomes

We prepared a RIP assay buffer consisting of PBS buffer (Gibco) and 10 mM MgCl_2_. To 17 μl of RIP assay buffer we added 2 μl of rabbit reticulocyte lysate (untreated) (Promega) or isolated ribosome and added purified RIP toxin at a final concentration of 5 μg/ml. All control samples were exposed to RIP storage buffer (50 mM Tris HCl, 250 mm NaCl, 212 mM imidazole, 1 mM EDTA, 10% glycerol, pH 8.9) containing no RIP toxin. Depurination assays ran at 30°C for 1 h and then were immediately placed on ice. Ribosomal RNA was extracted using the TRIzol (Invitrogen) extraction protocol and cDNA was produced using the SuperScript III reverse transcriptase (Invitrogen) protocol. The resulting cDNA was diluted 1:5 in nuclease-free dH_2_O and stored at −20°C to be used later for downstream analysis. We tested the stability of ribosomal RNA by incubating aliquots of isolated ribosomes in RIP assay buffer (1:1) at 30°C for 1 h. The ribosomes were then run on a 1% agarose gel and observed for any signs of degradation (Fig. S1).

A dosage curve was performed to compare depurination activities between native *Spiroplasma* RIP toxins and truncated *Spiroplasma* RIP toxins. Each reaction consisted of 17 μl of RIP assay buffer, 2 μl of rabbit reticulocyte, and 1 μl of RIP solution. The dosage curve consisted of 1:5 serial dilutions for each RIP toxin. Control samples were exposed to RIP storage buffer containing no RIP toxin. The dosage curve assays ran at 30°C for 15 min and then were immediately placed on ice. Ribosomal RNA was extracted using the TRIzol extraction protocol and cDNA was produced using the SuperScript III reverse transcriptase protocol. The resulting cDNA was diluted 1:5 in nuclease-free dH_2_O (Invitrogen) and stored at −20°C to be used later for downstream analysis. 10 mM MgCl_2_ was omitted from the RIP assay buffer used in the dosage curve experiments and in a supplementary depurination assay (Fig. S2). The results of these experiments are similar to earlier experiments performed under identical conditions where 10 mM MgCl_2_ was included. Therefore, the absence of 10 mM MgCl_2_ does not affect our interpretation of the results.

### RIP assays on live cells

The duration of RIP exposure was designed to extend for at least two generation cycles of each organism used in this study. *S. cerevisiae* cells in exponential growth phase were exposed to 1 μg/ml of each purified RIP toxin for 3 h at 30°C while shaking. *A. albopictus* cells were exposed to 1 μg/ml of each purified RIP toxin for 36 h at 28°C and 5% CO_2_. *Drosophila melanogaster* cells were exposed to 1 μg/ml of each purified RIP toxin for 48 h at 28°C and 5% CO_2_. *A. albopictus* and *D. melanogaster* cells were both cultured as semi-adherent monolayers and were grown to 80% confluence before being exposed to RIP toxins. In experiments involving truncated RIP toxins (i.e. Fig. 6A and C), live *D. melanogaster* cells were exposed to 1 μg/ml of each RIP toxin for 24 h at 28°C and 5% CO_2_, and live *S. cerevisiae* cells were exposed to 1 μg/ml of each RIP toxin for 3 h at 30°C while shaking. Control samples in all experiments were exposed to RIP storage buffer containing no RIP toxin. *S. cerevisiae* cells were pelleted at the end of RIP exposures and the supernatant was removed. The *S. cerevisiae* cell pellet was resuspended in TRIzol for RNA extraction. Culture media was removed from *A. albopictus* and *D. melanogaster* cells at the end of RIP exposures and the cells were rinsed with fresh culture media. The fresh culture media was removed, and the cells were resuspended in TRIzol for RNA extraction. Isolated RNA was converted to cDNA by following the SuperScript III reverse transcriptase protocol. The resulting cDNA was diluted 1:5 in nuclease-free dH_2_O and stored at −20°C to be used later for downstream analysis.

### qPCR-based analysis of RIP activity

RIP activity was measured using the cDNA products of RIP exposure experiments and a qPCR-based protocol developed by a previous study [37]. The protocol takes advantage of reverse transcriptase’s tendency to pair gaps in the RNA sequence (i.e. depurination site) with an adenine. Thus, the cDNA product of unmodified rRNA will have a thymine at the same position where the cDNA product of depurinated rRNA will have an adenine. This discrepancy in nucleotide identity is detectable by making the 3′-most nucleotide of qPCR primers complementary to the depurinated variant. The sensitivity of these primers was increased further by changing the third position from 3′ end to a nonmatching nucleotide except for the *Drosophila*-specific primers which were acquired from a previous study [27]. We also designed a set of control primers along the same contiguous strand of 28S rRNA to act as normalizers for downstream data transformation.

Fold changes in depurination were measured using the Pfaffl ratio method [38]. This approach subtracts the Ct of each sample from the global mean which allows us to plot control samples and RIP-treated samples together. Data were plotted in R version 4.2.0. A summary of the primers is available in Table S4. qPCR Ct values and data transformations are available in supplementary data.

### Phylogeny building

MAFFT 7.388 [39] was used to create an alignment of *Spiroplasma* RIP domains. RIP domains are flanked with accessory domains that are diverse and often nonhomologous. To exclude these uninformative regions from our phylogeny building, we trimmed sequences immediately outside of the conserved active site residues prior to alignment. MEGA software [40] was then used to determine the best model for PhyML tree building [41]. WAG + G + F parameters were used to build the RIP phylogeny (Fig. 1). All domains and intrinsically disordered protein regions (IDRs) of RIP-bearing proteins were annotated using HMMER 3.3 [42]. RIP accessions can be found in Table S5.

### Statistical analyses

Depurination assays were statistically compared with Tukey’s range test with *P-*value threshold < .001. Dosage curve assays were statistically compared with a two-way analysis of variance (ANOVA) with *P*-value threshold < .001. All RIP toxins were used in equal mass per volume concentrations (i.e. μg/ml) within assays. Mass per volume does not account for proteins of different lengths and therefore, the number of enzyme molecules (i.e. picomoles) in a reaction will vary between different RIP toxins within assays and may skew statistical outcomes. Reaction rates are linearly correlated with enzyme concentration when the substrate is in excess [43]. Given this, all RIP assays used in this study were converted from micrograms per milliliter to picomoles. RIP toxins were standardized for each assay and the depurination data were adjusted accordingly. All statistical analyses were performed again. Only data from RIP exposures to *A. albopictus* isolated ribosomes (Fig. 4B) and to live *S. cerevisiae* (Fig. 4D) exhibited a shift in significance groupings; however, this shift does not affect the interpretation of our results. Adjusted data and the outcomes of statistical analyses are available in supplementary data.

## Results

### *Spiroplasma* species encode multiple active RIP toxins

**Figure 3:**
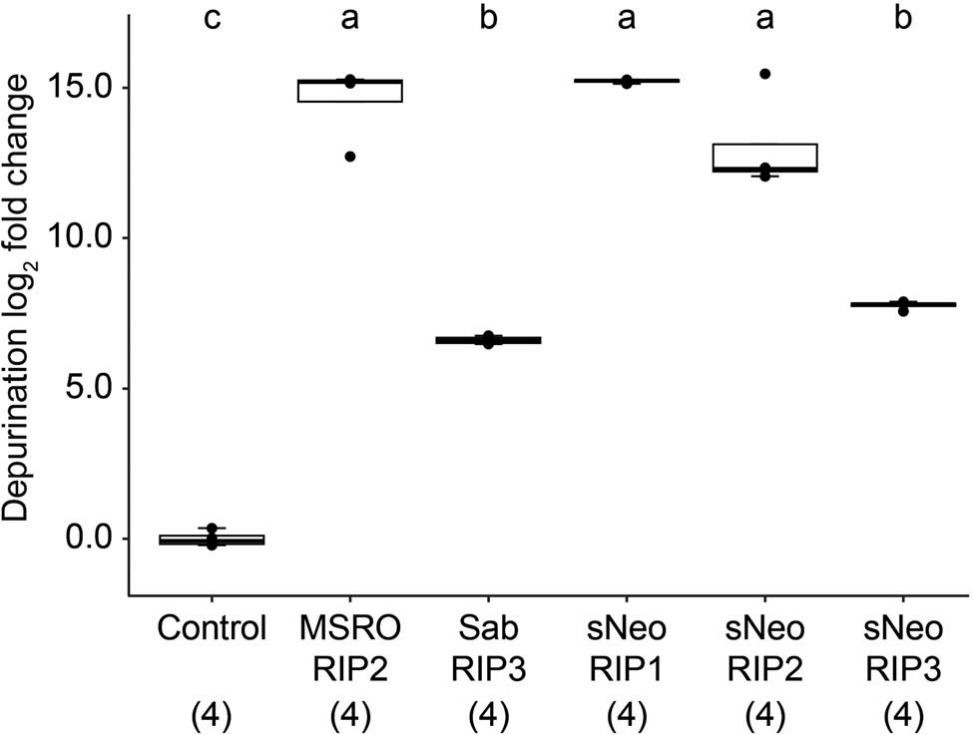
Depurination activity is retained across diverse RIP toxins. Five RIP toxins were sampled across their known diversity and tested on rabbit reticulocyte to determine if any retain enzymatic activity. All five RIPs are capable of depurinating ribosomes including three encoded by the same *Spiroplasma* strain (sNeo RIP1–3). Numbers beneath toxin names indicate sample size. *Y*-axis baseline adjusted to zero. Tukey test, *P* < .001. Different lowercase letters indicate statistically significant differences.

Using recombinant expression in *E. coli*, we purified RIP toxins derived from three *Spiroplasma* strains including three from the *Spiroplasma* symbiont of *D. neotestacea* (sNeo RIP1, sNeo RIP2, and sNeo RIP3), one from the *Spiroplasma* symbiont of *D. melanogaster* (MSRO RIP2) and one from *Spiroplasma sabaudiense* (Sab RIP3) (Fig. 1). *S. sabaudiense* is not known to be a vertically transmitted or defensive symbiont of insects but is recognized to infect mosquitoes with potential pathogenic effects [44]. RIP toxins display N-glycosidase (i.e. depurination) activity at a specific adenine residue in a conserved region of the 28S ribosomal RNA, the α-sarcin/ricin loop (SRL). RIP activity is measured in this study using a qPCR-based protocol to calculate the quantity of depurinated 28S rRNA relative to total 28S rRNA in RIP-exposed and control samples [37]. To first test whether the purified *Spiroplasma* RIPs exhibit N-glycosidase activity on eukaryotic 28S rRNA, we exposed each RIP to rabbit reticulocyte lysate, which includes freely accessible ribosomes. Results show that all five RIP toxins are enzymatically active and capable of N-glycosidase activity at the SRL (Fig. 3). These results confirm: (i) diverse and distantly related *Spiroplasma* RIP toxins retain their predicted enzymatic functions and (ii) individual *Spiroplasma* strains maintain multiple active RIP toxins (i.e. *Spiroplasma* strain *sNeo*). There are also notable differences in levels of depurination activity between different RIP toxins. sNeo RIP1 displays among the highest depurination activity against rabbit ribosomes and Sab RIP3 the lowest, with a nearly 400-fold difference between the two.

### *Spiroplasma* RIP toxins are active across diverse eukaryotic ribosomes

**Figure 4:**
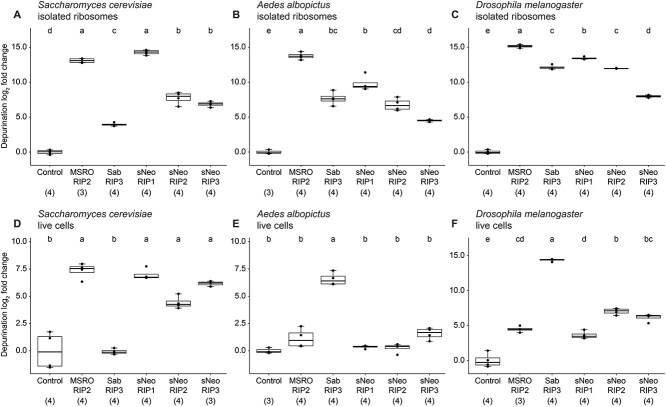
*Spiroplasma* RIP toxin activity is restricted based on ribosome and cell identity. Five RIP toxins were tested on ribosomes isolated from cell cultures of *S. cerevisiae*, *A. albopictus*, and *D. melanogaster*. RIP toxins were also exposed to live cells of *S. cerevisiae*, *A. albopictus*, and *D. melanogaster*. All RIP toxins tested are capable of depurinating all isolated ribosome types. Boxplots include (A) isolated *S. cerevisiae* ribosomes exposed to each RIP toxin, (B) isolated *A. albopictus* ribosomes exposed to each RIP toxin and, (C) isolated *D. melanogaster* ribosomes exposed to each RIP toxin. The depurination activity of *Spiroplasma* RIP toxins is influenced by the cell type they are exposed to. Boxplots include (D) live *S. cerevisiae* cells exposed to each RIP, (E) live *A. albopictus* cells exposed to each RIP, and (F) live *D. melanogaster* cells exposed to each RIP. Numbers beneath toxin names indicate sample size. *Y*-axis baseline adjusted to zero. Tukey test, *P* < .001. Different lowercase letters indicate statistically significant differences.

RIP activity was tested on three additional ribosome types including those isolated from *A. albopictus* (C7–10), *Saccharomyces cerevisiae* (HA0)*,* and *D. melanogaster* (S2). All five RIP toxins are active against all three eukaryotic ribosomes (Fig. 4A–C). Similar to rabbit ribosomes, *Spiroplasma* RIPs exhibit different levels of activity against *A. albopictus*, *S. cerevisiae,* and *D. melanogaster* ribosomes and hierarchies of activity vary by ribosome type. For example, sNeo RIP1 and MSRO RIP2 have similar activity when exposed to *S. cerevisiae* ribosomes, but MSRO RIP2 has significantly higher activity against ribosomes isolated from *A. albopictus* and *D. melanogaster* relative to all other toxins. Additionally, Sab RIP3 has the lowest activity against *S. cerevisiae* ribosomes, but Sab RIP3 activity is equivalent to or greater than the activity of other RIP toxins against *A. albopictus* and *D. melanogaster* ribosomes. Nucleotide identity is highly conserved across the SRLs present in this study and across domains of life with regards to the GAGA tetraloop regions and the G-bulge cross strand stack region (Fig. S3). However, nucleotide identity in the flexible region is divergent across species and this region is known to influence RIP activity in a ricin-based system [45].

### *Spiroplasma* RIP toxins demonstrate preference for cell types

To determine whether cellular barriers influence susceptibility to *Spiroplasma* RIPs, we exposed cells from the same culture lines that ribosomes were isolated from including *A. albopictus*, *S. cerevisiae*, and *D. melanogaster*. Whereas all five RIP toxins were capable of depurinating each type of isolated ribosome (Fig. 4A and C), exposure of live cells to RIPs significantly restricted depurination activity depending on the toxin (Fig. 4D and F). All RIP toxins are active against ribosomes of live *S. cerevisiae* cells except for Sab RIP3, which shows no significant depurination activity (Fig. 4D). Alternatively, the only RIP toxin with significant levels of depurination against the ribosomes of live *A. albopictus* cells is Sab RIP3 whereas all other RIP toxins show no significant activity (Fig. 4E). In *D. melanogaster* cells, Sab RIP3 is once again the most active toxin with depurination activity over 160-fold greater than the next most active toxin―sNeo RIP2 (Fig. 4F). Lastly, sNeo RIP1 and sNeo RIP2 were included in this study to examine potential functional differences between two relatively closely related RIPs encoded by the same symbiont strain. Despite their homology, the hierarchies of sNeo RIP1 and sNeo RIP2 depurination switch between cell types. sNeo RIP1 exhibits significantly higher depurination activity against *S. cerevisiae* cells (Fig. 4D) and sNeo RIP2 exhibits significantly higher depurination activity against *D. melanogaster* cells (Fig. 4F). Altogether, these results implicate the existence of barriers (e.g. cell membranes and cellular trafficking) that prevent specific RIP toxins from accessing and depurinating cytosolic ribosomes and suggest that some of the genetic diversity observed within the *Spiroplasma* RIP toxin gene family is relevant to navigating these barriers.

### Hyperdiverse accessory domains contribute to *Spiroplasma* RIP activity and specificity

The accessory domains are a striking source of diversity across *Spiroplasma* RIP toxins (Fig. 1) and are suspected of being involved in target specificity. However, nothing is known about the molecular interactions between these domains, the RIP toxin domain, and the cells that are targeted. We selected MSRO RIP2 and Sab RIP3 for further exploration into the influence of accessory domains on RIP toxin activity. MSRO RIP2 was selected for its high levels of depurination activity against isolated ribosomes (Fig. 4A and C), variable levels of depurination against live cells (Fig. 4D and F), and a prominent C-terminal accessory domain (Fig. 1). Sab RIP3 was selected because it exhibits the highest levels of depurination against live insect cells (Fig. 4E and F) and because it lags behind most other *Spiroplasma* RIP toxins when exposed to live yeast (Fig. 4D) and isolated ribosomes (Fig. 4A and C). Additionally, closer inspection reveals that the small N-terminal accessory region of Sab RIP3 encodes an IDR—a feature observed in several other *Spiroplasma* RIP toxins (Fig. S4).

**Figure 5:**
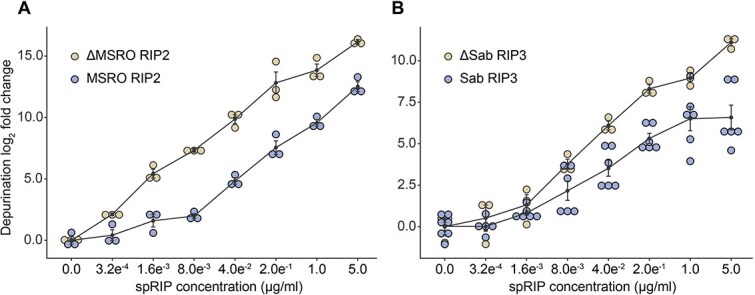
Removal of RIP accessory domains increases depurination activity. (A) A dosage curve showing the depurination activity of MSRO RIP2 and ΔMSRO RIP2 in 1:5 serial dilutions against rabbit reticulocyte. Truncating the C-terminal accessory domain of MSRO RIP2 results in a significant increase in depurination activity. *Y*-axis baselines adjusted to zero. *n* = 3 for each dosage concentration, two-way ANOVA, *P* < .001. (B) A dosage curve showing the depurination activity of Sab RIP3 and ΔSab RIP3 in 1:5 serial dilutions against rabbit reticulocyte. Truncating the N-terminal intrinsically disordered protein region results in a moderate and statistically significant increase in depurination activity. *Y*-axis baselines adjusted to zero. *n* = 3 for each dosage concentration of ΔSab RIP3 exposures and *n* = 6 for each dosage concentration of Sab RIP3 exposures, two-way ANOVA, *P* < .001.

The accessory regions of MSRO RIP2 and Sab RIP3 were removed via mutagenesis to produce ΔMSRO RIP2 (Fig. 3A) and ΔSab RIP3 (Fig. 3B). The depurination activity of truncated and nontruncated toxins were compared in a dosage-dependent RIP assay using rabbit reticulocyte. Results show that in both exposures, the truncated versions of the *Spiroplasma* RIP toxins exhibit higher levels of depurination than their native counterparts. For instance, between concentrations of 8 ng/ml and 200 ng/ml, ΔMSRO RIP2 depurination activity is upwards of 40-fold greater than MSRO RIP2 (Fig. 5A). At 5 μg/ml, ΔSab RIP3 depurination activity is over 20-fold greater than Sab RIP3 (Fig. 5B). These results demonstrate that the regions flanking the *Spiroplasma* RIP toxin domains can have an inhibitory effect on depurination activity.

**Figure 6:**
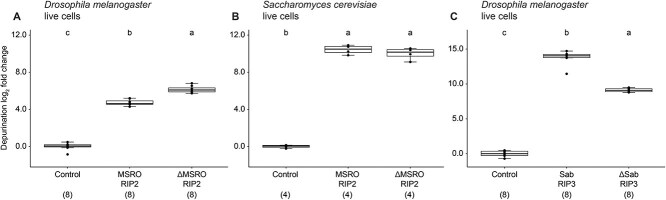
Removal of RIP accessory domains alters depurination activity against live cells. Live cells were exposed to two divergent RIP toxins with either their accessory domain intact or truncated. Removal of accessory domains influences RIP activity depending on cell and ribosome type. (A) Live *D. melanogaster* cells exposed to MSRO RIP2 or ΔMSRO RIP2. (B) Live *S. cerevisiae* cells exposed to MSRO RIP2 or ΔMSRO RIP2. (C) Live *D. melanogaster* cells exposed to Sab RIP3 or ΔSab RIP3. Numbers beneath toxin names indicate sample size. Baselines adjusted to zero. Tukey test, *P* < .001. Different lowercase letters indicate statistically significant differences.

We explored how the removal of accessory domains affect depurination of ribosomes in live cells. We exposed cell cultures of *S. cerevisiae* and *D. melanogaster* to ΔMSRO RIP2 and MSRO RIP2 and cell cultures of *D. melanogaster* to Sab RIP3 and ΔSab RIP3. Results revealed a two-fold increase in ribosome depurination activity in *D. melanogaster* cells by ΔMSRO RIP2 compared to MSRO RIP2 (Fig. 6A). This increase in depurination is statistically significant but is an order of magnitude less than the maximum increase observed by ΔMSRO RIP2 against free rabbit ribosomes. We found no significant difference in depurination activity against live *S. cerevisiae* between ΔMSRO RIP2 and MSRO RIP2 (Fig. 6B). In contrast, removal of the accessory domain from Sab RIP3 reduced depurination 25-fold in live *D. melanogaster* cell assays relative to intact Sab RIP3 (Fig. 6B). We confirmed with ΔSab RIP3 and Sab RIP3 exposures to isolated *D. melanogaster* ribosomes that this decrease in activity is not attributed to differences in interactions with the *Drosophila* ribosome itself (Fig. S2). Because ΔSab RIP3 was created by removing an N-terminal sequence of peptides that includes an IDR region, these results suggest that IDRs, which are a relatively common feature found in the accessory domains of diverse *Spiroplasma* RIP toxins (Fig. S4), may play an important role in facilitating RIP toxin access to cytosolic ribosomes.

## Discussion

Understanding how evolutionary processes and ecological context together shape the function of toxins remains a high priority for research across microbial and multicellular systems. Our study addresses this question in an insect symbiosis using the expanded RIP toxin repertoire of *Spiroplasma*. Using a phylogenetically informed experimental approach, we show that the sequence and structural diversity maintained in this gene family corresponds to differences in enzymatic activity *in vitro* and cellular targeting *in vivo*. These changes appear to have emphasized specificity over generality for some of the toxins we investigated here, enhancing RIP effectiveness in certain biological contexts and attenuating in others—an expected outcome of neofunctionalization following duplication. Although our data do not demonstrate these changes are or have been adaptive in ecological context, they highlight how diversity-generating processes including mutation, horizontal gene transfer, and recombination have facilitated the acquisition, modification, exchange, and loss of hypervariable domains likely to influence N-glycosidase activity and cellular access.

Our results suggest that natural selection to retain N-glycosidase activity has accompanied copy number expansion in the *Spiroplasma* RIP toxin family. The retention of multiple active RIP toxins in *sNeo* and other strains may suggest: (i) increased copy number has facilitated greater abundance of expressed toxin, or (ii) individual copies have specialized roles. The latter point is supported by past evidence suggesting specific involvement of sNeo RIP1 and/or sNeo RIP2 in defense against the parasitic nematode, *Howardula* [32]. The panel of purified RIP toxins also included MSRO RIP2 and Sab RIP3 which are encoded by the defensive *Spiroplasma* strain MSRO of *D. melanogaster* and by the possibly pathogenic *Spiroplasma* strain of *Aedes* mosquitoes, respectively. The presence of an active RIP toxin in *S. sabaudiense*, which is not known to be a vertically transmitted mutualistic strain, also implies *Spiroplasma* RIPs may perform functions unrelated to arthropod host defense. This observation may be relevant to the biology and pathogenesis of multiple RIP—encoding *Spiroplasma* pathogens [44, 46–48].

In order to exert their toxic effects, protein toxins may need to circumvent various structural or physiological barriers (e.g. the cell membrane, cell transport pathways, or target identity). These barriers may vary between cell types and can promote protein toxin diversification and specialization on those cell targets [49–51]. We tested whether ribosomal elements serve as one of these barriers for *Spiroplasma* RIPs by exposing isolated eukaryotic ribosomes to purified RIPs. All three ribosome types we tested—vertebrate, invertebrate, and fungal–were usable substrates for *Spiroplasma* RIPs, but some RIPs did display differential depurination activity across these targets. Specific interactions with ribosomes have been found to influence depurination in other RIP toxin systems. For instance, the SRL is a highly conserved region of 28S rRNA although sequence diversity is observed in the flexible region and these differences can influence RIP toxin depurination [45]. The flexible region is the only region of the SRL of ribosomes used in this study that exhibits nucleotide diversity (Fig. S3) and may be a partial barrier to RIP activity. Another ribosomal feature recognized to engage in specific interactions with RIP toxins is the P-stalk, which helps facilitate GTPase activity alongside the α-sarcin/ricin loop [52]. Both ricin and Shiga toxins are shown to engage in specific interactions with the P-stalk and these interactions are crucial to depurination activity [53, 54]. Although the SRL is a highly conserved target, the architecture of the P-stalk varies across eukaryotic organisms [55] and may also be a partial barrier to some *Spiroplasma* RIP toxins.

Despite our finding that all eukaryotic ribosomes tested in this study are vulnerable to RIP depurination activity, we show that ribosomes in live cells of those same organisms are not equally vulnerable; in some cases, depurination of intracellular ribosomes is prevented entirely depending on the toxin and target pair (Fig. 4). In contrast, some RIP toxins depurinated cellular ribosomes far more effectively than others, suggesting they can circumvent cellular barriers or engage in specific interactions to overcome them. These results are consistent with *Spiroplasma* defensive capabilities where RIP toxin activity varies by symbiont strain and natural enemy identity [26, 27]. Altogether, these observations support the hypothesis that neofunctionalization is a common outcome in *Spiroplasma* RIP toxin expansion and that their diversification may in part be driven by the selective benefit of targeting new and diverse cell types. However, cell targets for future assays will need to consider the various cell types that exist within the same multicellular organism following development. For instance, cells cultures from *D. melanogaster* and *A. albopictus* are derived from embryonic and neonate larva, respectively [56, 57]. It is possible that RIP toxin accessibility will change as tissue cells become more specialized and diverse throughout development.

RIP toxicity depends on their ability to enter cells. Type 2 RIP toxins, like ricin, consist of two peptide chains connected by a single disulfide bond—an A-chain encoding the toxic RIP domain and a B-chain encoding a sugar-binding lectin domain [58, 59]. Alternatively, type 1 RIP toxins (e.g. all *Spiroplasma* RIP toxins) consist of only the A-chain but may still display efficient cytosolic access in systems other than those known to be ecological targets of defensive *Spiroplasma* [60]. Our results support that the hyperdiverse accessory domains flanking RIP domains are at least partially involved in these molecular processes. We find that removal of the accessory domains from the divergent RIP toxins MSRO RIP2 and Sab RIP3 results in a significant increase in depurination levels, suggesting type 1 RIP accessory domains may have an intrinsically inhibitory effect. Some non*Spiroplasma* RIP toxins, including both ricin and Shiga toxin, are inactive while the A-chain remains in complex with the accessory B-chains due to the B-chain blocking the A-chain’s ribosome binding sites or active sites [61, 62]. Separation of the A-chain from the holotoxin is a crucial part of both ricin and Shiga toxin trafficking [63, 64], and modification by the host environment or machinery is a common theme in bacterial protein toxin activation. Separation of toxin domains from accessory subunits is a typical and necessary step in many of these cases [9, 64–66]. In this context, our results raise questions over the fate of some *Spiroplasma* RIP toxin accessory domains following internalization and trafficking within eukaryotic cells, especially given the potential benefit of their dissociation.

*Spiroplasma* RIP toxins cause collateral damage to their *Drosophila* hosts [34]. However, the negative fitness outcomes of this damage are far less severe than what targeted parasites sustain (i.e. death and sterility) [27–32]. Given that these toxins are secreted freely into the host hemolymph, *Spiroplasma* RIP toxins must actively avoid host cells while targeting parasite cells. MSRO RIP2 is encoded by *Spiroplasma* strain MSRO which is a natural symbiont of *D. melanogaster*. MSRO RIP2 exhibits the highest levels of depurination against isolated *D. melanogaster* ribosomes but among the lowest levels of depurination against live *D. melanogaster* cells (Fig. 4). This difference in potential RIP activity (i.e. activity against isolated ribosomes) and actual RIP activity (i.e. activity in live cells) suggests defensive toxins like MSRO RIP2 possess features that minimize collateral damage against the host. One such feature may be the accessory domain, as revealed by our truncation experiments. For instance, removal of the MSRO RIP2 accessory domain more than doubles the amount of depurination sustained by live *D. melanogaster* cells (Fig. 6). Altogether, these data suggest that *Spiroplasma* limits toxin-mediated collateral damage to the host and that accessory domains facilitate this by minimizing localization within host cells and reducing RIP activity on host ribosomes.

Sab RIP3 is one of the smallest and most divergent RIP toxins encoded by *Spiroplasma*. The RIP domain makes up >70% of the entire protein and no recognizable signal peptide is present (Fig. 1). However, Sab RIP3 displays high levels of depurination against live cultured insect cells relative to other toxins. There is limited research on the relationship between *S. sabaudiense*, the species that encodes Sab RIP3, and mosquito hosts. Past experiments have shown that *S. sabaudiense* causes cytotoxicity when co-cultured with *Aedes* cells [44]. In another study, intrathoracic injections of *S. sabaudiense* into *Aedes* mosquitoes resulted in reduced progeny with a sex ratio bias towards males [67]. One notable feature of Sab RIP3 is the presence of an IDR region located N-terminally to the RIP domain. IDRs lack a stable tertiary conformation, and have been implicated in multiple roles especially related to molecular binding [68]. Exposures of both Sab RIP3 and ΔSab RIP3 against live *Drosophila* cells reveal that the removal of the IDR—possessing accessory domain results in a significant decrease in depurination activity against those cells.

IDR regions are associated with diverse protein toxins. Among the most well-studied are the toxins bearing the RTX motifs that transition from disordered to ordered in the presence of Ca+ [69]. Although present on a variety of toxins with ranging biological contexts, RTX motifs appear to often be involved in secretion from the host bacteria although with exceptions. A particularly well-studied example is the CyaA toxin encoded by *Bordetella pertussis*—the causative agent of whooping cough. The RTX motifs of CyaA are crucial to binding with target cell membrane proteins for accessing the cytosol [70]. IDR regions are also frequent features in virus proteomes [71]. Virtually all viruses encode proteins with IDR regions, many of which are identified to be involved in host manipulation. IDR regions appear to be relatively common across *Spiroplasma* RIP toxins including sNeo RIP1, sNeo RIP2, and MSRO RIP2 (Fig. S4). These regions will be strong candidates for future investigations into *Spiroplasma* RIP toxin specificity.

## Conclusion

In this study, we demonstrate the functional diversity of ribosome-targeting toxins encoded by bacterial symbionts in the genus *Spiroplasma*. Our results using cell-free ribosomes demonstrate that RIP activity has been conserved within and between *Spiroplasma* strains amid toxin family expansion and diversification. In contrast, our cell culture assays reveal that only a subset of RIPs are capable of reaching and inactivating ribosomes inside each of the live cell types tested. Together, these results support the hypothesis that both of the features required to support parasite defense, i.e. toxicity and specificity, can be encoded within a single protein. Our truncation experiments show that: (i) toxicity is encoded within the predicted N-glycosidase domain, (ii)accessory domains inhibit rather than enhance ribosome inactivation, and (iii) for the toxin in our panel with specificity for insect cells, specificity is reduced by removing the N-terminal accessory domain. These results suggest that the hyperdiverse accessory domains of RIPs have the potential to serve the dual roles needed for defensive toxins to minimize host harm while effectively targeting parasites. There are three groups of parasites that *Spiroplasma* symbionts are known to defend against―nematodes [31], parasitoid wasps [28, 72], and fungi [73]. The observed phylogenetic diversity of RIP toxins may suggest *Spiroplasma* defends against additional natural enemies or that parasite resistance to *Spiroplasma* has repeatedly facilitated the emergence and persistence of novel RIP variants. The ribosome and cell panels used in the current study allowed characterization of fundamental features of *Spiroplasma* RIPs, but are limited in comparison to the variety of potential targets that may contribute to shaping toxin diversity in natural populations. Future work will extend these studies to additional target organisms, especially those with ecological relevance to hosts of RIP-encoding *Spiroplasma* strains.

## Supplementary Material

Supplementary_materials_wraf174

Supplementary_data_wraf174

## Data Availability

The datasets generated during and/or analysed during the current study are available in the supplementary data and in the supplementary tables.
