# Supplementary material for: Evolution of specialized toxin arsenals in a bacterial symbiont of arthropods
Source: ISME J. 2025 Aug 11;19(1):wraf174. doi: 10.1093/ismejo/wraf174 (PMC12422007; doi:10.1093/ismejo/wraf174)
Supplement: Supplementary_materials_wraf174 [file supplementary_materials_wraf174.pdf]

Supplementary figures

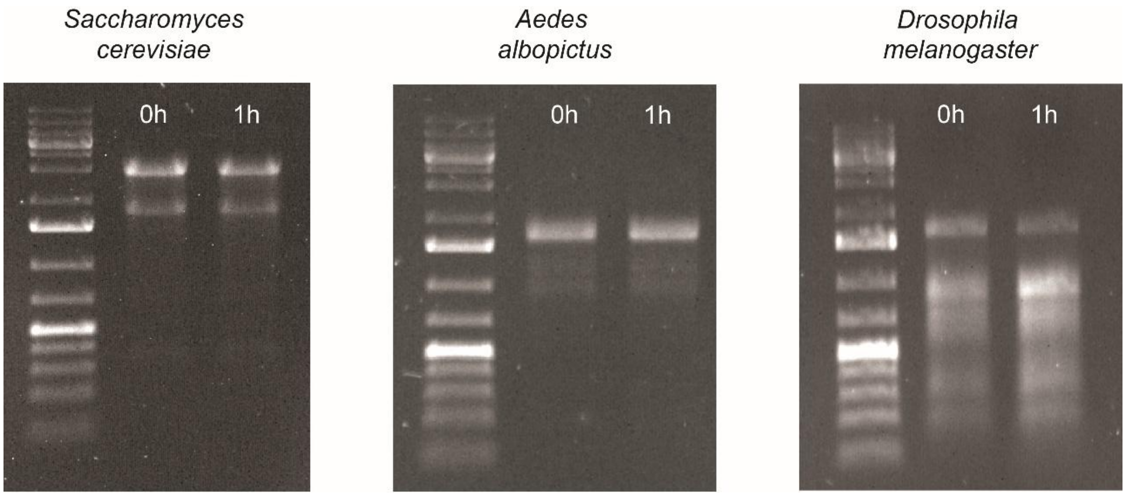

Figure S1. Isolated ribosomes are stable under RIP exposure conditions. Isolated ribosomes were mixed 1:1 with Gibco PBS buffer + 10 mM Mg_2Cl and incubated for zero hours (0h) or incubated at 30°C for one hour (1h) or mixed. Ribosomes then ran on a 1% agarose gel alongside an unincubated control to detect degradation of rRNA. Yeast and mosquito ribosomal rRNA is stable during incubation. *D. melanogaster* ribosomes exhibit small amounts of degradation but this does not appear to affect depurination.

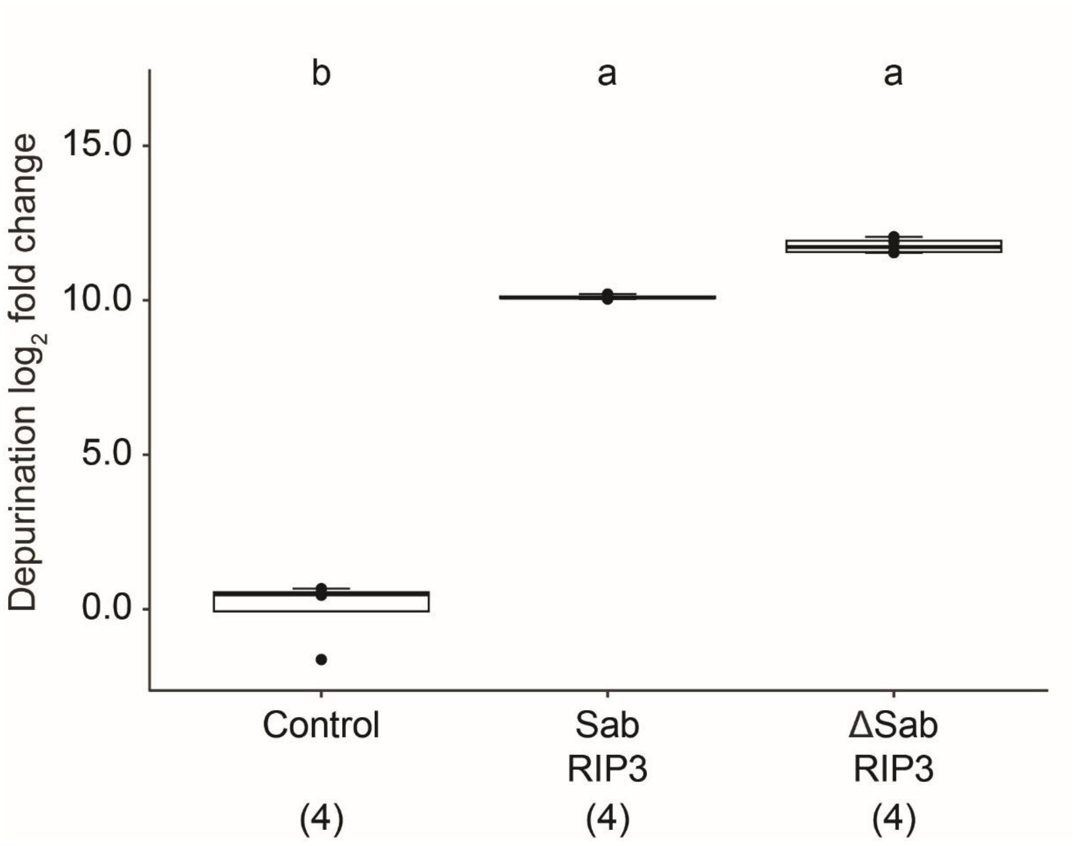

Figure S2. Sab RIP3 and ΔSab RIP3 exhibit equal levels of depurination against isolated *Drosophila* ribosomes. Sab RIP3 and ΔSab RIP3 were exposed to isolated *D. melanogaster* ribosomes. These results suggest that the differences in depurination activity against live *Drosophila* cells is not due to the properties of *Drosophila* ribosomes. To 17 µl of RIP assay buffer we added 2 µl of isolated *Drosophila* ribosome and added purified RIP toxin at a final concentration of 5 µg/ml. Depurination assays ran at 30°C for 1 hour. Numbers beneath toxin names indicate sample size. Y-axis baseline adjusted to zero. Tukey test, $P < 0.001$. Different lower-case letters indicate statistically significant difference.

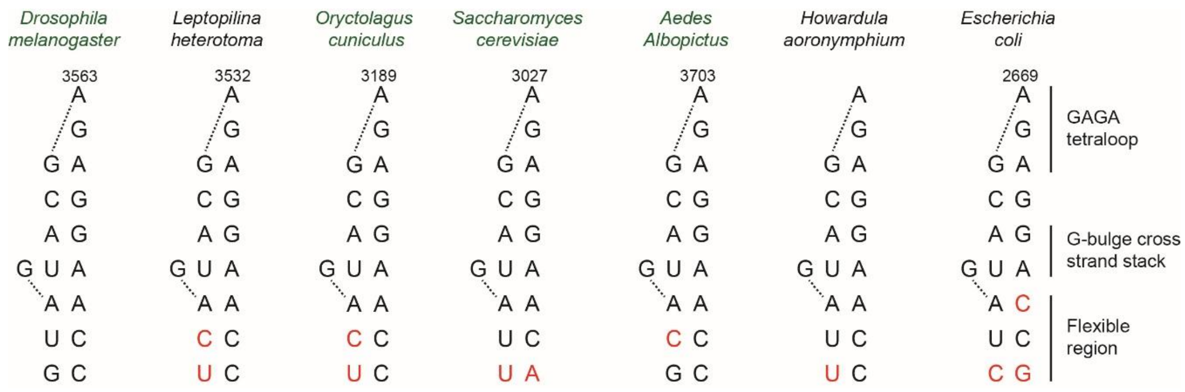

Figure S3. Nucleotide conservation in the α-sarcin/ricin loop. α-sarcin/ricin loops of various ribosome types known to be targets of *Spiroplasma* RIP toxins or used in this study. Accessions for each SRL are listed in Table S1. *Howardula aoronymphium* 28S was assembled in this study from an SRA read file. The names of organisms tested in this study are shown in green text. Nucleotide identities that deviate from *Drosophila melanogaster* SRL are shown in red text.

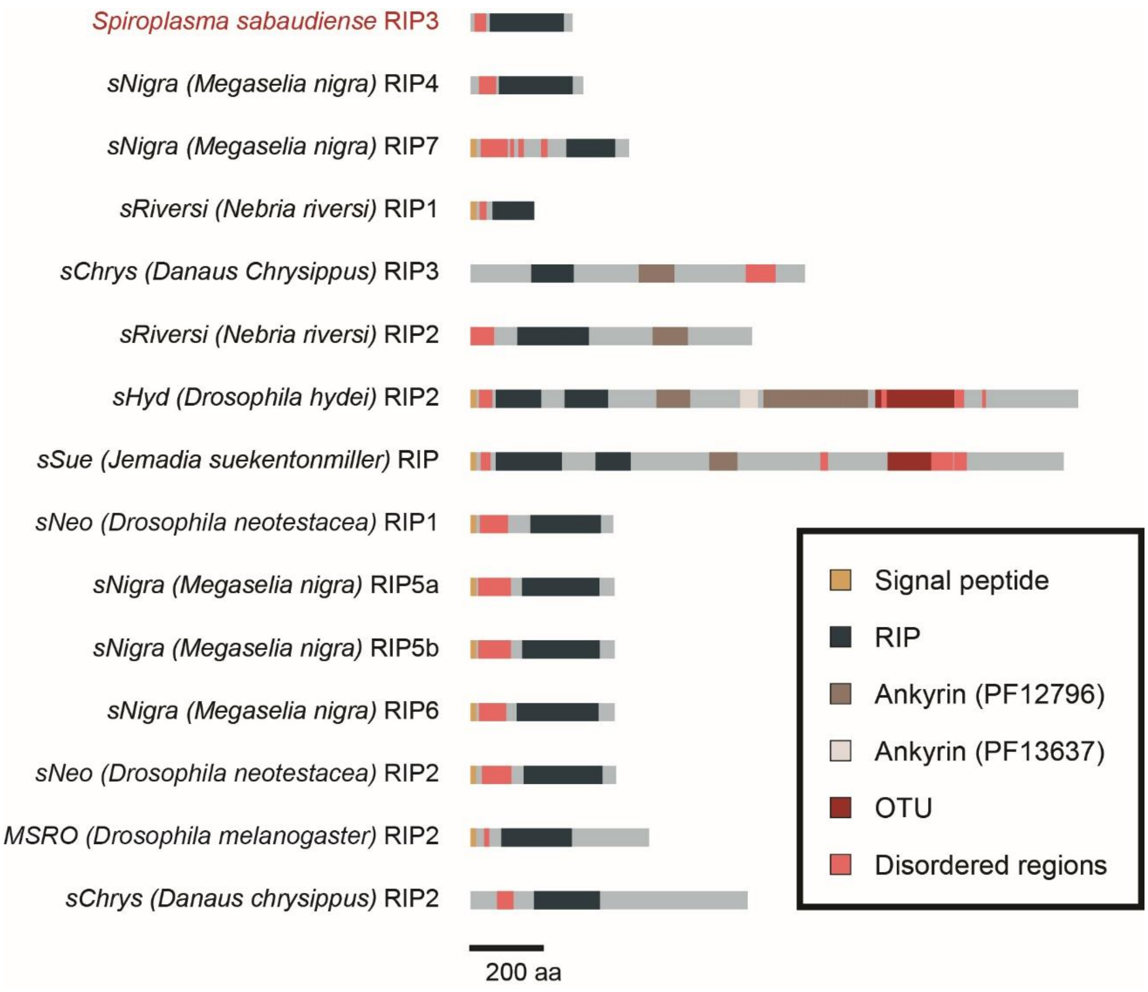

Figure S4. Disordered regions are a common feature of *Spiroplasma* RIP toxins. A scaled representation of all *Spiroplasma* RIP toxins with predicted intrinsically disordered protein regions. Sab RIP3 (labeled in red text) shows evidence of specific interactions with live cells and has a predicted disordered region on the N-terminal side of the RIP domain. Removal of this region significantly attenuates depurination by Sab RIP3 in live cells. Only disordered regions ≥10 amino acids in length are shown.

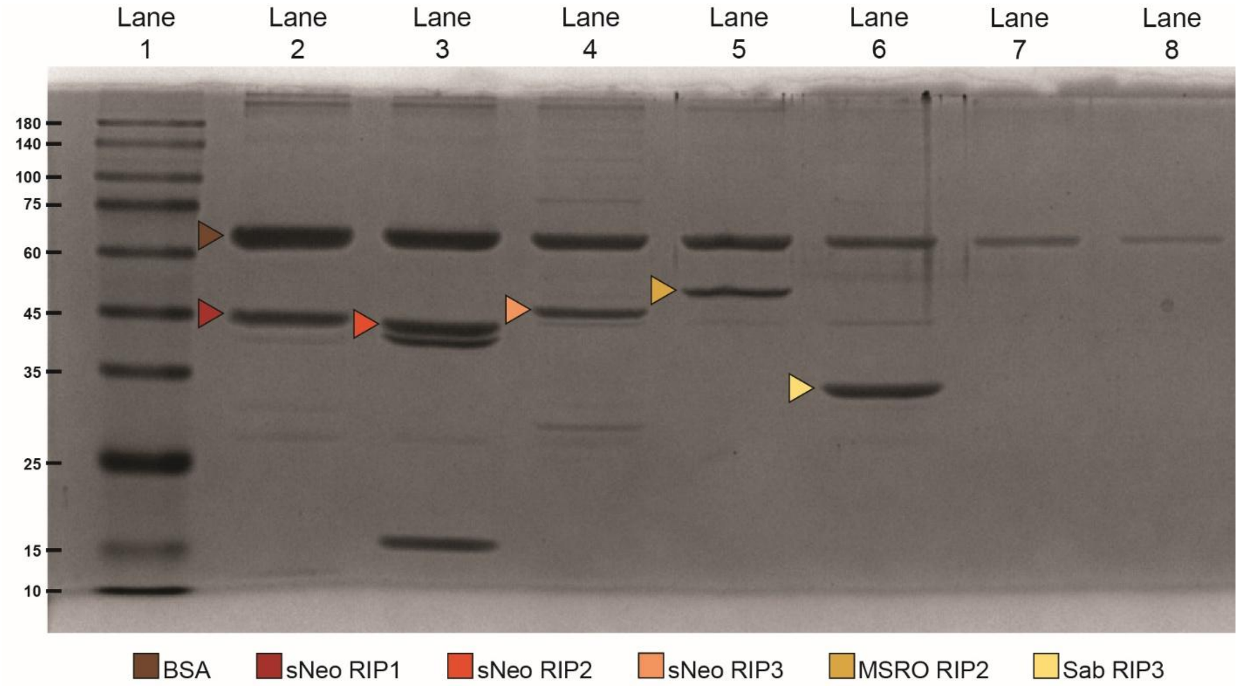

Figure S5. SDS-PAGE gel of purified RIP toxins and BSA standard. The contents of each lane are as follows: Lane 1) PM2500 ExcelBand 3-color Regular Range Protein Marker, Lane 2) sNeo RIP1 elute + 2.000 µg Bovine Serum Albumin (BSA), Lane 3) sNeo RIP2 elute + 1.500 µg BSA, Lane 4) sNeo RIP3 elute + 1.000 µg BSA, Lane 5) MSRO RIP2 elute + 0.750 µg BSA, Lane 6) Sab RIP3 elute + 0.500 µg BSA, Lane 7) 0.250 µg BSA, and Lane 8) 0.125 µg BSA. The added volumes of each RIP elute were adjusted to ensure the total amount of each RIP would fall within the BSA standard curve and thus, ensure accurate quantitation.

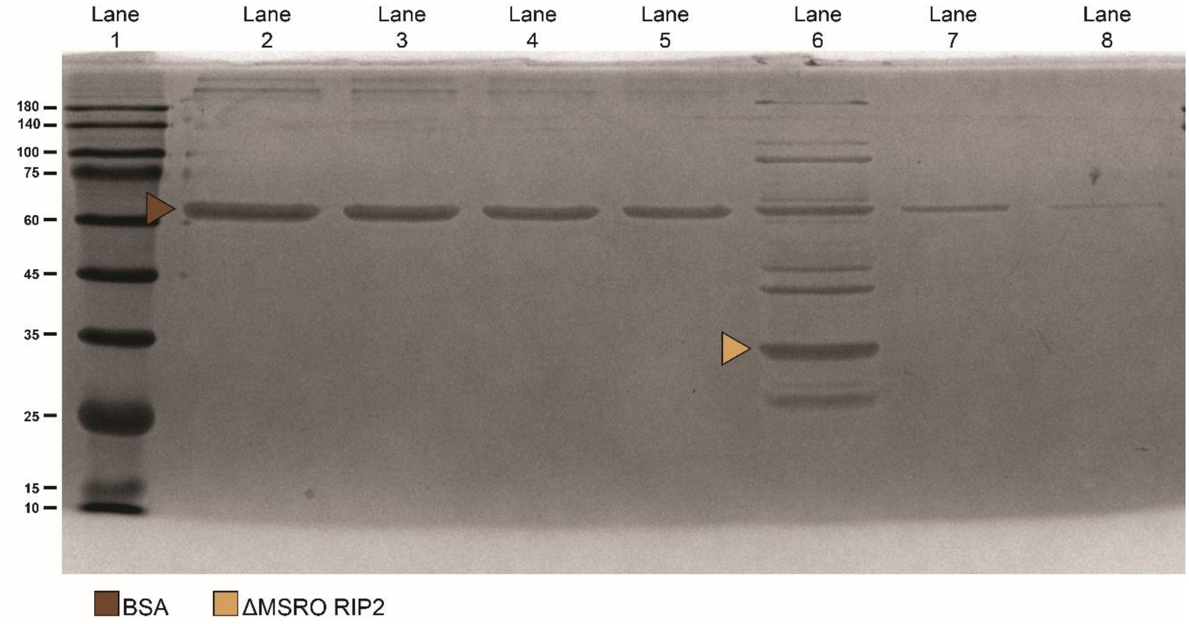

Figure S6. SDS-PAGE gel of purified ΔMSRO RIP2 and BSA standard. The contents of each lane are as follows: Lane 1) PM2500 ExcelBand 3-color Regular Range Protein Marker, Lane 2) 2.000 μg Bovine Serum Albumin (BSA), Lane 3) 1.500 μg BSA, Lane 4) 1.000 μg BSA, Lane 5) 0.750 μg BSA, Lane 6) ΔMSRO RIP2 + 0.500 μg BSA, Lane 7) 0.250 μg BSA, and Lane 8) 0.125 μg BSA.

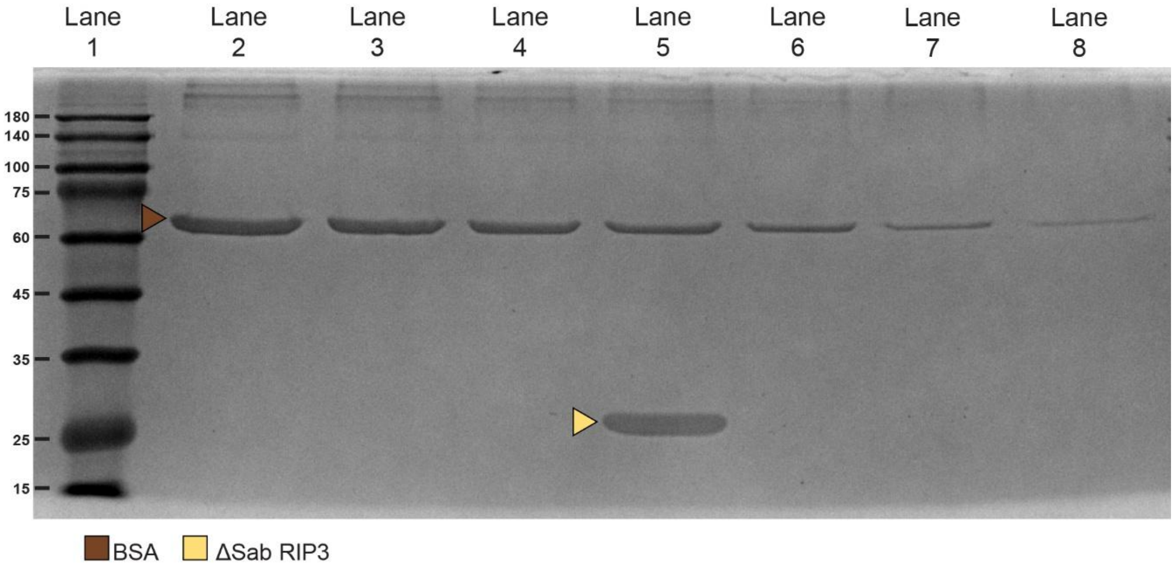

Figure S7. SDS-PAGE gel of purified ΔSab RIP3 and BSA standard. The contents of each lane are as follows: Lane 1) PM2500 ExcelBand 3-color Regular Range Protein Marker, Lane 2) 2.000 µg Bovine Serum Albumin (BSA), Lane 3) 1.500 µg BSA, Lane 4) 1.000 µg BSA, Lane 5) ΔSab RIP3 0.750 µg BSA, Lane 6) 0.500 µg BSA, Lane 7) 0.250 µg BSA, and Lane 8) 0.125 µg BSA.

Supplementary tables

Table S1 Ribosomal rRNA accessions for SRLs shown in Figure 3D

Organism	Accession
Drosophila melanogaster	Gene ID: 26067172
Leptopilina heterotoma	Gene ID: 122507271
Oryctolagus cuniculus	Nucleotide accession: 7TOR_A28S; 1-3552 bp
Sacchromyces cerevisiae	Nucleotide accession: CP006468; 433,444 - 436,839 bp
Aedes albopictus	Gene ID: 115265929
Howardula aeronymphium	SRA accession: SRR10825152
Escherichia coli	Nucleotide accession: NZ_CP014272; 201,190 - 204,114 bp

Table S2 Molecular properties of purified RIP toxins

Toxin	Molecular Weight	Total elute (%)	Amount
sNeo RIP1	43.95 kDa	91.61%	0.59 µg/µl
sNeo RIP2	42.12 kDa	43.14%	0.21 µg/µl
sNeo RIP3	45.00 kDa	76.96%	0.10 µg/µl
MSRO RIP2	49.70 kDa	100%	0.34 µg/µl
ΔMSRO RIP2	33.25 kDa	37.7%	0.26 µg/µl
Sab RIP3	32.54 kDa	92.87%	0.55 µg/µl
ΔSab RIP3	25.83 kDa	100%	1.59 µg/µl

Table S3 Summary of primers used for Q5 Site-Directed Mutagenesis

Primer sets	Sequences
ΔSab RIP3	5' ACG CCC GAA TTT GAA G 3'<br>5' CAT CTA GTA TTT CTC CTC TTT C 3'
ΔMSRO RIP2	5' GAG AAT TTA TAT TTT CAA GGA CAC 3'<br>5' CTC CGG CAG ATT AAA G 3'

Table S4 Summary of qPCR primers used to detect depurination

Primer sets	Sequences	R^2	Efficiency
S. cerevisiae Depurinated	$^{5'}$GCG TTG GAT TGT TCA CCC AC $^{3'}$<br>$^{5'}$CCA ATT ATC CGA ATG AAC TGT TCC AC**A** $^{3'}$	.99	89.4%
S. cerevisiae Normalizer	$^{5'}$GGG CTC ATG GAG AAC AGA AAT CTC $^{3'}$<br>$^{5'}$GAT CGA TAG GCC ACA CTT TCA TGG $^{3'}$	.99	93.4%
A. albopictus Depurinated	$^{5'}$TAA CGG AAA TGC TGT GCA GTA TG**T** $^{3'}$<br>$^{5'}$CGA CCT TAG AGG CGT TCA GG $^{3'}$	.99	97%
A. albopictus Normalizer	$^{5'}$CGT TCA GTC ATG TCG TCA TTC CTG $^{3'}$<br>$^{5'}$AGT ATC CAG ATG TCT TAC TGT CAT CGG $^{3'}$	.99	84.5%
Rabbit Depurinated †	$^{5'}$CAT GGT AAT CCT GCT CAG TAT G**T** $^{3'}$<br>$^{5'}$ATT CTG ACT TAG AGG CGT TCA GTC $^{3'}$	.99	96.7%
Rabbit Normalizer †	$^{5'}$CTA AAC CAT TCG TAG ACG ACC T $^{3'}$<br>$^{5'}$TTG TGT CGA GGG CTG ACT T $^{3'}$	.99	95.7%
D. melanogaster Depurinated ‡	$^{5'}$CGA CAG CAT TCC TGC GTA GTA AG**T** $^{3'}$<br>$^{5'}$ACA ATG CAA ATT GCC CCT TA $^{3'}$	.99	90.8%
D. melanogaster Normalizer ‡	$^{5'}$GTT CCA ATT CCG TAA CCT GTT GAG $^{3'}$<br>$^{5'}$GTC ATG CTC TTC TAG CCC ATC T $^{3'}$	.99	89.5%

† primers were acquired from Hamilton et al., 2016
‡ primers were acquired from Ballinger et al., 2017

Table S5 Accessions for RIP toxins shown in Figure 1

RIP Queries	RIP Accessions
E. coli Shiga toxin Subunit A	WP_062860140
sAtri (*Drosophila atripex*) RIP 1	WDA54629.1
sAtri (*Drosophila atripex*) RIP 2, 3	WDA53903.1
MSRO (*Drosophila melanogaster*) RIP 1	WP_040093770
MSRO (*Drosophila melanogaster*) RIP 2	WP_040093936
MSRO (*Drosophila melanogaster*) RIP 3, 4, 5	WP_126821432
sChrys (*Danaus chrysippus*) RIP 1	WP_174479925
sChrys (*Danaus chrysippus*) RIP 2	WP_174479900
sChrys (*Danaus chrysippus*) RIP 3	WP_174481319
sCinc (*Cephus cinctus*) RIP	TLF25546
sCoccus (*Dactylopius coccus*) RIP	MBP1525224
sDis (*Lariophagus distinguendus*) RIP	WP_252319696
sHyd (*Drosophila hydei*) RIP 1	JXYY01000068; 2633-4054 bp
sHyd (*Drosophila hydei*) RIP 2 *spaid-like*	JXYY01000041; 4552-9630 bp
sMoj (*Drosophila mojavensis*) RIP 1	MBH8624287
sNeo (*Drosophila neotestacea*) RIP 1	WP_127093322
sNeo (*Drosophila neotestacea*) RIP 2	ASM46792
sNeo (*Drosophila neotestacea*) RIP 3	ASM46791
sNeo (*Drosophila neotestacea*) RIP 4	WP_158676203
sNigra (*Megaselia nigra*) RIP 1	WP_126821049
sNigra (*Megaselia nigra*) RIP 2	WP_126821476
sNigra (*Megaselia nigra*) RIP 3	WP_126821462
sNigra (*Megaselia nigra*) RIP 4	WP_126821293
sNigra (*Megaselia nigra*) RIP 5a,b	WP_126821432
sNigra (*Megaselia nigra*) RIP 6	WP_126820955
sNigra (*Megaselia nigra*) RIP 7	WP_126821022
Spiroplasma eriocheiris RIP 2	WP_047791253
Spiroplasma eriochieris RIP 1	WP_079450805
Spiroplasma mirum RIP 1	WP_025317327
Spiroplasma sabaudiense RIP 2	WP_025251436
Spiroplasma sabaudiense RIP 3	WP_025250933
Spiroplasma sabaudiense RIP1	WP_025251437
sRiversi (*Nebria riversi*) RIP 1	WP_215826391
sRiversi (*Nebria riversi*) RIP 2	WP_215825920
sRubra (*Myrmica rubra*) RIP	LJ130441.1, LJ130442.1
sScab (*Myrmica scabrinodis*) RIP	SAMN08120314; Contig 25; 10916-12094 bp
sSue (*Jemadia Suekentonmiller*) RIP	DWDU01007497; 333-5285
sVan (*Myrmica vandeli*) RIP	SAMN08120315; Contig 24351; 424-977 bp

Supplementary Methods

Purification of sNeo RIP1, sNeo RIP2, sNeo RIP3, and Sab RIP3

Five *Spiroplasma* RIP toxins were selected for recombinant expression and purification including sNeo RIP1-3, Sab RIP3, and MSRO RIP2 (Figure 1). Signal peptides were detected using SignalP 6.0 and removed at the predicted cleavage site. The RIP sequences underwent codon optimization for expression in *E. coli*. A TEV cleavage site and a 6-His tag was added to the C-terminal end of sNeo RIP1, sNeo RIP2, sNeo RIP3, and Sab RIP3. These gene constructs were then inserted into a pBR322 plasmid. SHuffle T7 Express Competent *E. coli* were transformed with these RIP-bearing plasmids. Cultures were grown to 0.400-0.600 OD600 and expression was induced with 1 mM IPTG (final conc.) (Sigma-Aldrich) for 4 hours at 30°C. Following induction, the culture was spun down and the cell pellet was lysed in B-PER lysis buffer (Thermo Scientific) following the manufactures protocol. The cell lysate then spun down and the supernatant was removed, leaving an inclusion body pellet which was resuspended in 500 µL of equilibration buffer [50 mM Tris HCl (Research Products International), 250 mM NaCl (Fisher Scientific), 10 mM imidazole (Sigma-Aldrich), pH 8.9 for all RIP toxins except sNeo RIP3 which was pH 9.5). SDS (Tokyo Chemical Industry) was added to a final concentration of 1% and the inclusion body suspension was sonicated with a probe sonicator five times for 5 seconds each. Following sonication, Triton-X 100 (Integra Chemical) was added to a final concentration of 2% and the mixture was spun on an end-over-end rotator for an hour at 4°C. The cell lysis was then transferred to 5 mL of equilibration buffer containing 1X Halt Protease Inhibitor Cocktail (Thermo Scientific) and spun on an end-over-end rotator overnight at 4°C. The incubated lysis was incubated with HisPur Ni-NTA Resin (Thermo Scientific) for an hour on an end-over-end rotator at 4°C. The resin was rinsed twice with wash buffer (50 mM Tris HCl, 250 mM NaCl, 20 mM imidazole, pH 8.9 for all RIP toxins except sNeo RIP3 which was pH 9.5) and eluted with elution buffer (50 mM Tris HCl, 250 mM NaCl, 212 mM imidazole, pH 8.9 for all purified RIP toxins). Elutions were measured with a nanodrop to estimate protein concentration. The highest elutions were combined with a final concentration of 1 mM EDTA (Research Products International) and 10% glycerol, and stored at -80°C. For molecular analysis, purified toxins were run on an SDS-PAGE gel with a BSA standard (EMD Millipore) ranging from 2.0 mg/ml to 0.125 mg/ml. Protein concentration, molecular weight and purity were determined using Bio-Rad Image Lab software. SDS-PAGE of purified and summary statistics of sNeo RIP1-3 and Sab RIP3 are available in supplementary materials (Figure S5 and Table S2).

Purification of MSRO RIP2, ΔMSRO RIP2, and ΔSab RIP3

MSRO RIP2 required a different expression approach due to difficulties expressing in *E. coli*. Like the previous RIP toxins, the N-terminal signal peptide was removed at the predicted cleavage site and the gene was optimized for expression in *E. coli*. A TEV cleavage site and a 6-His tag was added to the N-terminal end and an additional 6-His tag was also added to the C-terminal end. The gene construct was inserted into a pET-15b plasmid already equipped with T7 expression regulatory sequences. SHuffle T7

Express Competent *E. coli* were transformed with the MSRO RIP2-bearing pET-15b plasmid as well as a pLysS plasmid which encodes T7 lysozyme for tighter control of expression.

Truncated versions of MSRO RIP2 (*ΔMSRO RIP2*) and Sab RIP3 (ΔSab RIP3) were created using the Q5 Site-directed mutagenesis kit (New England Biolabs) (Primers listed in Table S3). The toxin domains of MSRO RIP2 and Sab RIP3 were annotated with HHPRED software, and these annotations were confirmed with alignments to other *Spiroplasma* RIP toxins. In the case of ΔSab RIP3, 6 amino acids were removed from the N-terminal end of the RIP domain (predicted by HHPRED) however these amino acids were not conserved among other *Spiroplasma* RIP toxins. Therefore, the removal of these accessory domains is unlikely to have directly affected catalysis of either RIP toxin. Primers were designed to remove the region immediately downstream of the annotated MSRO RIP2 RIP domain and immediately upstream of the C-terminal His-tag (Figure 7A). The boundaries of the IDR region of Sab RIP3 were determined using HMMER software and primers were designed to remove the IDR region and all upstream material up to the start codon (Figure 7B). The ΔSab RIP3 open reading frame was then inserted into a pET-15b plasmid for more efficient expression. SHuffle T7 Express Competent *E. coli* were transformed with the ΔMSRO RIP2-bearing pET-15b plasmid along with the pLySs plasmid or with just ΔSab RIP3-bearing pET-15b. Cultures were grown to 0.400-0.600 OD600 and expression was induced with 1 mM IPTG (final conc.) for 4 hours at 30°C. Following induction, the cultures were spun down and the supernatant was removed. For *E. coli* expressing MSRO RIP2 or ΔSab RIP3, the cell pellet was lysed in B-PER lysis buffer following the manufactures protocol. For *E. coli* expressing ΔMSRO RIP2, the B-PER steps were omitted, and the entire cell pellet was immediately suspended in equilibration buffer. SDS was added to final concentrations of 1% and the suspensions were sonicated five times for 5 seconds. The remaining steps were performed as described for all other RIP toxins except the overnight spin was omitted for purification of ΔSab RIP3. Summary statistics of purified MSRO RIP2, ΔSab RIP3, and ΔMSRO RIP2 can be found in Table S2. SDS-PAGE of MSRO RIP2, ΔMSRO RIP2, and ΔSab RIP3 are available in supplementary materials (Figure S5-7).

Isolation of *Aedes albopictus* and *Drosophila melanogaster* ribosomes

The following protocol is adapted from previously described protocols [1,2]. Insect cells including *A. albopictus* (C710) and *D. melanogaster* (S2) were grown to 80% confluence across 6-8 culture plates. Cells were scraped and spun down at 6000xg for 10 minutes. The supernatant was removed, and the cells were resuspended in cell lysis buffer [50 mM Tris HCl, 10 mM $MgCl_2$ (Acros Organics), 150 mM NaCl, 2 U/ml Turbo DNase (Thermo Scientific), 10 U/ml SUPERase In (Thermo Scientific), 1X Halt protease inhibitor, 1 mM DTT (Invitrogen), pH 7.5]. The cell lysate was sonicated five times for five second bursts and the lysate cooled on ice for 60 seconds between each sonication step. The cell lysate was clarified at 20’000xg for 30 minutes at 4°C. The resulting supernatant was overlaid on a sucrose cushion buffer [50 mM Tris HCl, 10 mM $MgCl_2$,

150 mM NaCl, 10 U/ml SUPERase In, 1X Halt protease inhibitor, 1 mM DTT, 1 M sucrose (Agrose Organics), pH 7.5] at a 1:1 ratio and spun at 235'000xg for 4 hours at 4°C. Following centrifugation, the supernatant was removed, and the ribosome pellet was rinsed twice with ribosome wash buffer (50 mM Tris HCl, 10 mM $MgCl_2$, 150 mM NaCl, 1 mM DTT, pH 7.5). The ribosome pellet was resuspended in ribosome storage buffer (50 mM Tris HCl, 10 mM $MgCl_2$, 150 mM NaCl, 10 U/ml SUPERase In, 1X Halt protease inhibitor, 1 mM DTT, 10% glycerol, pH 7.5) and stored at -80°C.

Isolation of *Saccharomyces cerevisiae* ribosomes

The following protocol is adapted from previously described protocols[1–3]. *Saccharomyces cerevisiae* cells (HA0) were grown to 0.400 OD600. Cells were resuspended in cell lysis buffer and added to an equal volume of 0.5 mm glass beads. The bead slurry was agitated in a bead beater four times for 45 seconds and placed on ice for 60 seconds in between bead beating. The cell lysate was removed and brought up to 5 ml with cell lysis buffer. The cell lysate was then clarified at 20'000xg for 30 minutes at 4°C. The resulting supernatant was overlaid on a sucrose cushion buffer at a 1:1 ratio and spun at 235'000xg for 4 hours at 4°C. Following centrifugation, the ribosome pellet was rinsed twice with ribosome wash buffer and then resuspended in ribosome storage buffer and stored at -80°C.

References

1. Anger AM, Armache J-P, Berninghausen O *et al*. Structures of the human and Drosophila 80S ribosome. *Nature*; 2013; **497**: 80–5.

2. Rivera MC, Maguire B, Lake JA. Isolation of Ribosomes and Polysomes. *Cold Spring Harb Protoc*; 2015; **2015**: pdb.prot081331.

3. Fernández IS, Bai X-C, Hussain T *et al*. Molecular Architecture of a Eukaryotic Translational Initiation Complex. *Science*; 2013; **342**: 1240585.
